# Consensus Clustering and Survival-Related Genes of Cuproptosis in Cutaneous Melanoma

**DOI:** 10.1155/2023/3615688

**Published:** 2023-02-27

**Authors:** Wentao Qin, Fu Gan, Yiji Jike, Mingyang Jiang, Ang Li

**Affiliations:** ^1^Department of Orthopaedics, Zhongnan Hospital of Wuhan University, Wuhan, China; ^2^Department of Urology Surgery, The Affiliated Hospital of Youjiang Medical University for Nationalities, Baise, China; ^3^Department of Bone and Joint Surgery, The First Affiliated Hospital of Guangxi Medical University, Nanning, China; ^4^Emergency Center, Zhongnan Hospital of Wuhan University, Wuhan, China

## Abstract

As a highly malignant tumor, the morbidity and mortality of cutaneous melanoma (CM) are increasing year by year. A novel type of cell death connected to mitochondrial metabolism is called cuproptosis. Cuproptosis regulates tumor biological behavior. Thus, genes controlling cuproptosis could be a promising candidate bioindicator for cancer therapy. Datasets of CM patients were obtained from the public database that includes clinical information and RNA-seq data. We divided CM patients into three different subgroups by unsupervised clustering method and explored the differences in functional pathways among the three subgroups by GSVA to prove the possible potential mechanism of copper death-related genes in the formation and development of CM. Secondly, we used differential analysis and Cox regression analysis to find the differential genes related to prognosis, constructed the CRG score, found the critical score for dividing high and low CRG score groups, and then analyzed the prognosis and immune infiltration of high and low CRG score groups. The results show a great correlation between OS and CRG scores. Compared with patients with high CRG scores, patients with low CRG scores have a significantly higher survival rate. In a word, copper sagging plays a certain role in the progress of CM.

## 1. Introduction

Cutaneous melanoma (CM) develops from the malignant transformation of melanopoietic cells in the basal layer of the epidermis as a highly malignant tumor [1, 2]. Melanoma makes up only 5% of skin malignancies but is responsible for more than 75% of skin cancer fatalities [3]. It has become the 15th most common cancer worldwide and is a grave threat to humans' lives [4, 5]. Ultraviolet radiation is a major risk element for melanoma skin cancer [6, 7]. Ultraviolet radiation induces high mutation rates per megabase in somatic cells, and DNA damage signals play an important role in melanoma development [8]. MAPK pathway is the key cell signaling pathway in regulating cell growth, proliferation, and survival. In the treatment of melanoma, inhibition of the MAPK pathway plays an important role, and the research on inhibitors of MAPK pathway has made significant progress in recent years [9]. More than half of all melanoma patients express mutant BRAF, leading to constitutive activation of MEK and ERK signaling. Over the past ten years, patients with localized or metastatic melanoma have seen significant improvements in survival with immunotherapy and targeted therapies [10]. Although the new drugs in this pathway have achieved amazing clinical results, some people still have drug resistance, and the benefits in CM patients with BRAF mutation are likely to be temporary [11]. However, the drug is highly toxic, and efficacy varies from patient to patient due to the short duration of treatment [12]. The advanced IL-1-targeted therapy has been successfully used in mouse melanoma model, but it has not been applied to humans [13]. Therefore, the management of CM remains an evolving and challenging problem [14]. It is crucial to continue researching specific CM processes to enhance patients' long-term prognoses.

The human body requires copper, a trace metal, which is crucial for all living things' survival. Through antiangiogenesis, reactive oxygen species accumulation, and proteasome inhibition, copper can induce cellular autophagy or apoptosis [15]. As an essential cofactor, copper is mainly involved in three important processes in organisms: mitochondrial respiration, antioxidant defense, and biocompound synthesis. Cuproptosis is a relatively novel method of cell death that has been linked to mitochondrial metabolism [16]. It happens due to the lipid acylated tricarboxylic acid (TCA) cycle component's direct binding to copper. As a result, lipid acylated proteins begin to aggregate and iron-sulfur cluster proteins start to disappear. This causes proteotoxic stress and a particular kind of cell death [17]. Bian et al. thoroughly examined the genetic alterations in genes linked to copper toxicity in clear cell carcinoma of the kidney. They discovered that a worse overall survival (OS) was strongly connected with a higher risk score of the CRG signature. The progression-free survival was noticeably shorter in those at higher risk (PFS) [18]. Wu et al. thoroughly investigated the clinical and molecular features and the activity of cuproptosis regulators in 33 cancers using pan-cancer analysis. They discovered that cuproptosis was connected to a number of tumor-related pathways and immunological signals [19]. Besides, copper levels were much greater in cancer patients' serum and tumor tissue than in healthy ones [20]. Thus, interfering with copper homeostasis becomes a new target for cancer therapy. Cuproptosis translational medicine is not only used for genetic diseases, but more importantly may become a potential for cancer treatment [21]. Therefore, it is important to analyze the expression pattern and prognostic value of CRGs in the CM patients.

Here, we systematically analyzed the characteristics of CRG in CM based on TCGA and GEO RNA-seq datasets and clinical information. In addition, the predictive value of CRG in CM was clarified, and the predictive effect of CRG on immunotherapy efficacy in CM patients was evaluated. This provides a basis for clinical diagnosis, personalized immune targeted therapy, and antitumor drug resistance of CM patients.

## 2. Methods

### 2.1. Acquisition and Processing of CM Data

The Cancer Genome Atlas (TCGA) provided 471 CM samples along with clinical data, single nucleotide polymorphism (SNP), transcriptome RNA sequences (FPKM value), copy number variation (CNV), and transcriptome RNA sequences (SNP). For the combined study, 214 CM samples' transcriptome RNA sequences and clinical data were included in the GSE65904 cohort from the GEO. We thoroughly searched the available articles for CRGs and found CRGs with a variety of functions. We converted the FPKM value for CM samples obtained from the TCGA into TPM value using the “limma” tool in the R program. It was then combined with the GSE65904 cohort and standardized for additional genomic analysis and variant characterization [22]. Similar to this, CM samples from TCGA were merged and normalized with 812 normal tissue samples from UCSC Genome Browser for a further investigation of the differential expression of CRGs.

### 2.2. Clustering Analysis of CRGs

Unsupervised clustering was utilized to divide CM patients into subclusters based on the CRGs expressed in CM patients. Additionally, the clustering was carried out using the “ConsensusClusterPlus” R package. We distinguished subclusters according to the method of maximal intergroup and minimal intragroup variances. Furthermore, the distribution of CRGs was presented as a heat map in the three subclusters.

### 2.3. Gene Set Variation Analysis (GSVA)

The GSVA method was used to evaluate the biologically significant functions and regulatory mechanisms that genes might control [23]. Besides, we analyzed the functional pathways predominantly enriched in three CRG subclusters using the GSVA algorithm.

### 2.4. Differential Expressed Genes (DEGs) Linked to CRG Subclusters

With an updated *P* < 0.001 criteria, we screened DEGs in the three CRG subclusters using the “limma” tool (R software). Key biological activities of DEGs were then labeled and visualized using the GO [24] function and KEGG [25] enrichment analyses.

### 2.5. A Cluster Analysis Using Prognostic DEGs

We first employed a univariate Cox regression model to find the DEGs linked to prognosis in CM patients. Additionally, 352 DEGs related to prognosis were found in total. The Kaplan-Meier technique was then used to compare OS among gene subclusters after CM patients were separated into various gene subclusters based on prognosis-related DEGs by unsupervised clustering. Additionally, DEG heat maps were created between various gene subclusters.

### 2.6. Construction of the CRG Score

According to the expression of prognosis-associated DEGs, scores were obtained for each CM patient using principal component analysis (PCA). First, the CRG features A and B were determined as the DEGs that were favorably and negatively linked with the clustering features. The data were then further reduced in dimension using PCA. Principal components 1 and 2 were finally used to calculate the CRG feature scores: CRG score = ∑PC1_*i*_ + ∑PC2_*j*_. Each patient's CRG score was obtained, and then to categorize CM patients as having high or low CRG scores, we employed the “surv cutpoint” function. We then compared the OS of the two groups and chose the best cutoff. Additionally, we used the R package “limma” to further evaluate the disparity in CRG scores between CRG clusters and DEG clusters.

### 2.7. Analysis of the Relationship between the CRG Score and Clinical Features

Using the R package “maftools,” we examined the somatic mutation features of patients. Additionally, the most frequent mutation genes were displayed [26]. Patients were then divided into high and low TMB groups. We used the R package “survival” to examine the OS of the high and low CRG score groups in various genders and ages to investigate further the variations in CRG score across clinical variables.

### 2.8. CRG Score with Immunotherapy

Patients with malignant melanoma can see a considerable improvement in OS with immunotherapy and targeted treatments. First, we observed the variations of the immune checkpoint blocking (ICB) genes in the low and high CRG score groups [27, 28]. We validated the CRG score in predicting immunotherapy response in two cohorts. The GSE93157 cohort (*n* = 65) comprises patients with non-small-cell lung cancer, head and neck squamous cell carcinoma, and melanoma undergoing immunotherapy. IMvigor210 (*n* = 298) is an anti-PD-L1 immunotherapy cohort for patients with uroepithelial carcinoma.

### 2.9. Statistical Analysis

The R package's “survminer” was used for the analysis. The Kruskal-Wallis test and the Wilcox test were employed to compare two groups and more than two groups. The K-M technique and log-rank test were applied for survival analysis and survival difference statistics, respectively. All data analyses used R software version 4.0.2. Statistical significance was defined as a *P* value of 0.05 [29].

## 3. Results

### 3.1. Genetic and Variational Landscape of CRG in CM

**Figure 1:**
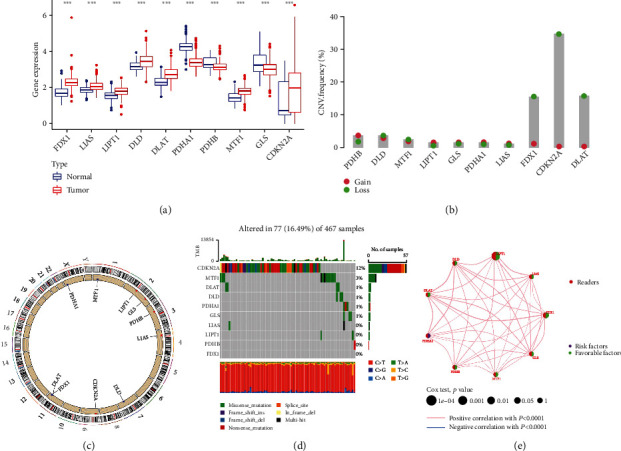
The genetic makeup of CRG variation in CM and its landscape. (a) The expression of 10 CRGs in normal skin tissues compared to that of CM tissues. Normal tissues are shown in blue, while malignant tissues are shown in red (^∗∗∗^*P* < 0.001). (b) The frequency of variations in the CRG copy number. A gain is denoted by the color red, whereas a loss is denoted by the color green. (c) Variation in the copy number of the CRG at various places on the chromosome. The color red indicates that the sample with the increased copy number is larger than the sample with the loss, and it is indicated by the color blue that the sample with the loss is larger than the increase. (d) A waterfall plot depicts the levels of CRG mutations. (e) Diagnostic and prognostic network for the CRGs. Red: “erasers”; orange: “readers”; gray: “writers”; CRG: cuproptosis-related gene; CM: cutaneous melanoma.

We merged the RNA sequences of 812 normal skin tissues from the UCSC and melanoma samples collected from the TCGA to investigate potential alterations in the expression of CRGs in normal tissues and cancer samples. The outcome (*P* < 0.05) is displayed in Figure 1(a). We then looked into the CNV of CRGs in CM by intersecting CM's gene CNV frequency data with the 10 CRGs. The frequency of copy number losses in the CRGs was higher than the frequency of gains in CM, with losses of CDKN2A, FDX1, and DLAT being noticeably higher than gains (Figure 1(b)). The outcomes of the CRGs with the loss and gain of copy numbers on various chromosomes are depicted in Figure 1(c). The top 2 genes with mutation frequency were CDKN2A (12%) and MTF1 (3%) (Figure 1(d)). Additionally, we utilized the univariate Cox survival analysis and K-M survival technique to understand the potential relationship between genes connected to CRGs and the OS of CM patients after deleting CRGs with zero expression. The CRG prognostic coexpression network's findings demonstrated a favorable correlation between the interactions between cuproptosis genes and prognosis, with LIPT1 significantly linked to prognosis in CM patients (Figure 1(e)).

### 3.2. Consensus Clustering Based on CRGs

**Figure 2:**
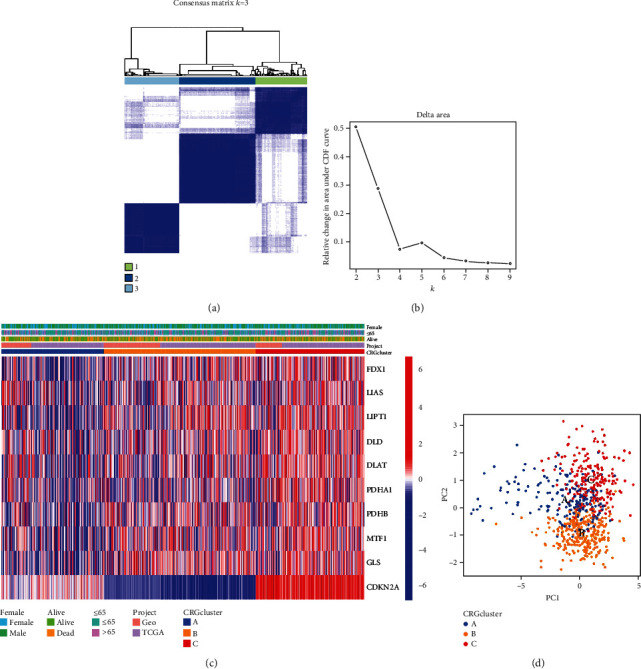
Clustering analysis based on the expression of CRGs. (a) Clustering according to a consensus at *K* = 3. (b) The relative change in the area under the CDF curve for the values of *K* ranges from 2 to 9. (c) A heat map of the CRG and its various clinical aspects, organized into three subclusters of the CRG. (d) PCA under the CRG modification pattern. CRG: cuproptosis-related gene; PCA: principal component analysis.

We divided CM into various molecular subclusters based on the consensus expression of cuproptosis regulators to explore probable underlying mechanisms of genes associated with cuproptosis in the formation and progression of CM. We used the “ConsensusClusterPlus” tool in the R programming language to group CM patients into several clusters. When *K* = 3, the clusters have little crossing between them and are closely connected internally (Figures 2(a) and 2(b)). The cuproptosis cluster's heat map showed that genes associated with the condition were most highly expressed in cluster C and least highly expressed in cluster A (Figure 2(c)). We discovered that the levels of CRGs may, in fact, separate CM patients into three distinct molecular subclusters after using PCA to identify changes in gene expression among the aforementioned three molecular subclusters (Figure 2(d)).

### 3.3. GSVA between Various Molecular Subclusters of Cuproptosis

**Figure 3:**
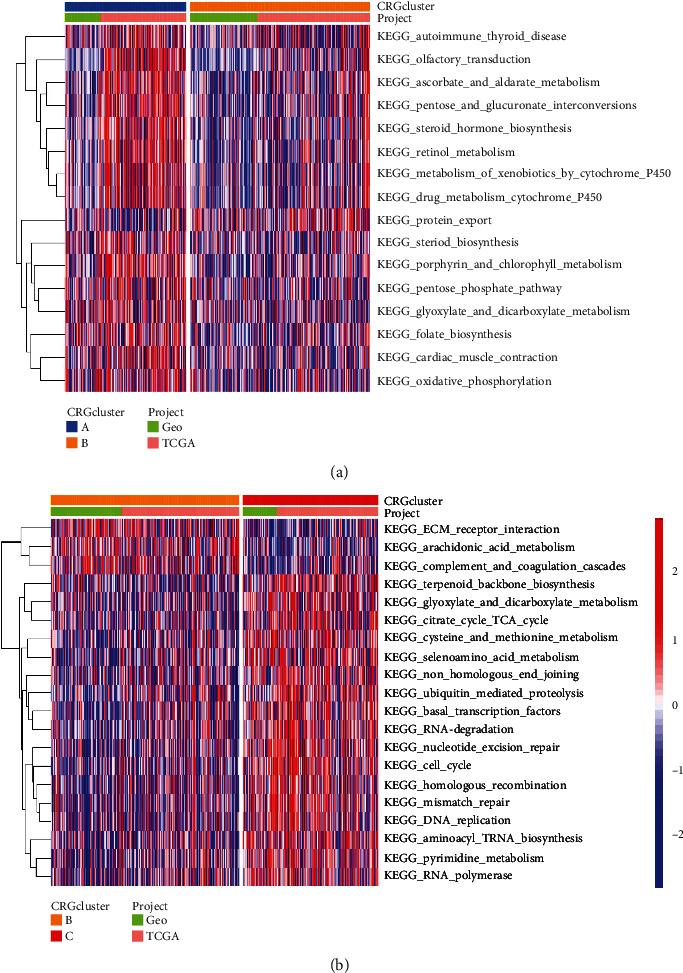
GSVA between distinct CRG subclusters. (a) An examination of the GSVA between clusters A and B. (b) An examination of the GSVA between clusters B and C. GSVA: gene set variation analysis; CRG: cuproptosis-related gene.

To comprehend probable molecular regulatory routes between various cuproptosis clusters of CM, GSVA was used for the three cuproptosis clusters. According to the comparison between clusters A and B, cluster A is primarily focused on ascorbate and aldarate metabolism, olfactory transduction, and autoimmune thyroid disease, while cluster B is focused on protein export (Figure 3(a)). Comparing clusters B and C, it was found that cluster C had active terpenoid backbone biosynthesis, glyoxylate and dicarboxylate metabolism, and citrate and TCA cycles (Figure 3(b)).

### 3.4. DEGs in Three Cuproptosis Subclusters

**Figure 4:**
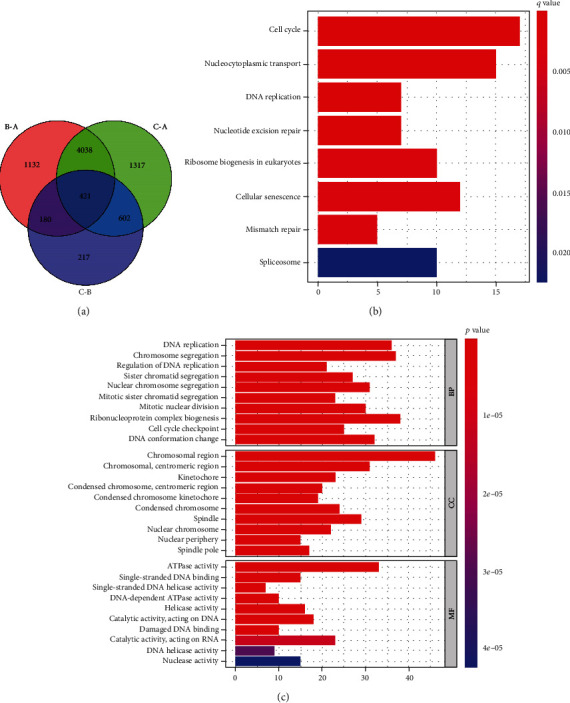
DEG identification and annotation of functions. (a) The Venn diagram of intersecting genes between CRGs. (b) An examination of the enrichment of DEGs in KEGG pathways. (c) An investigation of the enrichment of GO functions in DEGs. DEGs: differentially expressed genes; CRG: cuproptosis-related gene.

We set the criterion *P* value to screen the DEGs among the various subclusters to look into the potential biological activities of the various subclusters. 421 DEGs in all were tested (Figure 4(a)). These DEGs primarily participate in the cell cycle, nucleocytoplasmic transport, DNA replication, and nucleotide excision repair, according to the findings of the KEGG enrichment analysis (Figure 4(b)). Additionally, under GO enrichment analysis (Figure 4(c)), DEGs are primarily enriched in DNA replication, chromosome segregation, and control of DNA replication at the biological process (BP). Additionally, cell composition analysis (CC) results showed that the chromosomal region, chromosome, centromeric region, and kinetochore were enriched in the aforementioned DEGs. On the other hand, molecular function (MF) fractionation revealed that DEGs contribute to ATPase activity, single-stranded DNA binding, and DNA helicase activity.

### 3.5. Construction of the Subclusters of DEGs

**Figure 5:**
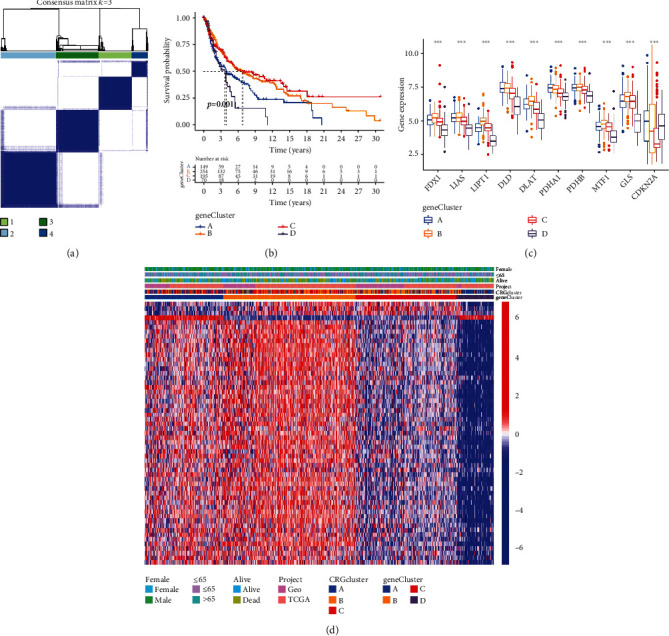
Consensus clustering analysis based on DEGs. (a) DEGs were used for consensus clustering, and the optimal *K* value was determined to be 4. Patients were divided into four subclusters. (b) K-M survival curves between four DEG subclusters. (c) Cuproptosis gene expression levels in four DEG subclusters. (d) The heat map of DEGs and the various clinical characteristics found in the four DEG subclusters. DEGs: differentially expressed genes; K-M: Kaplan-Meier.

We employed an unsupervised clustering approach to building a hierarchical clustering assessment of the expression profiles of prognostic associated DEGs to investigate more thoroughly the interactions and consistency among DEGs. When *K* = 4, there was the least crossover between subclusters and the strongest connectivity inside subclusters (Figure 5(a)). Besides, DEG cluster C had the longest OS (Figure 5(b)). Additionally, a boxplot was developed to show the expression of CRG within the context of the various gene clusters. Eight CRGs were expressed at higher levels in cluster B, while DLD and PDHA1 were expressed at significantly higher levels in cluster A, as shown in Figure 5(c). According to heat maps based on several DEG subclusters and various clinical traits, the cluster with the highest level of DEG expression was cluster B, followed by clusters A, C, and D (Figure 5(d)).

### 3.6. Constructing the CRG Score

**Figure 6:**
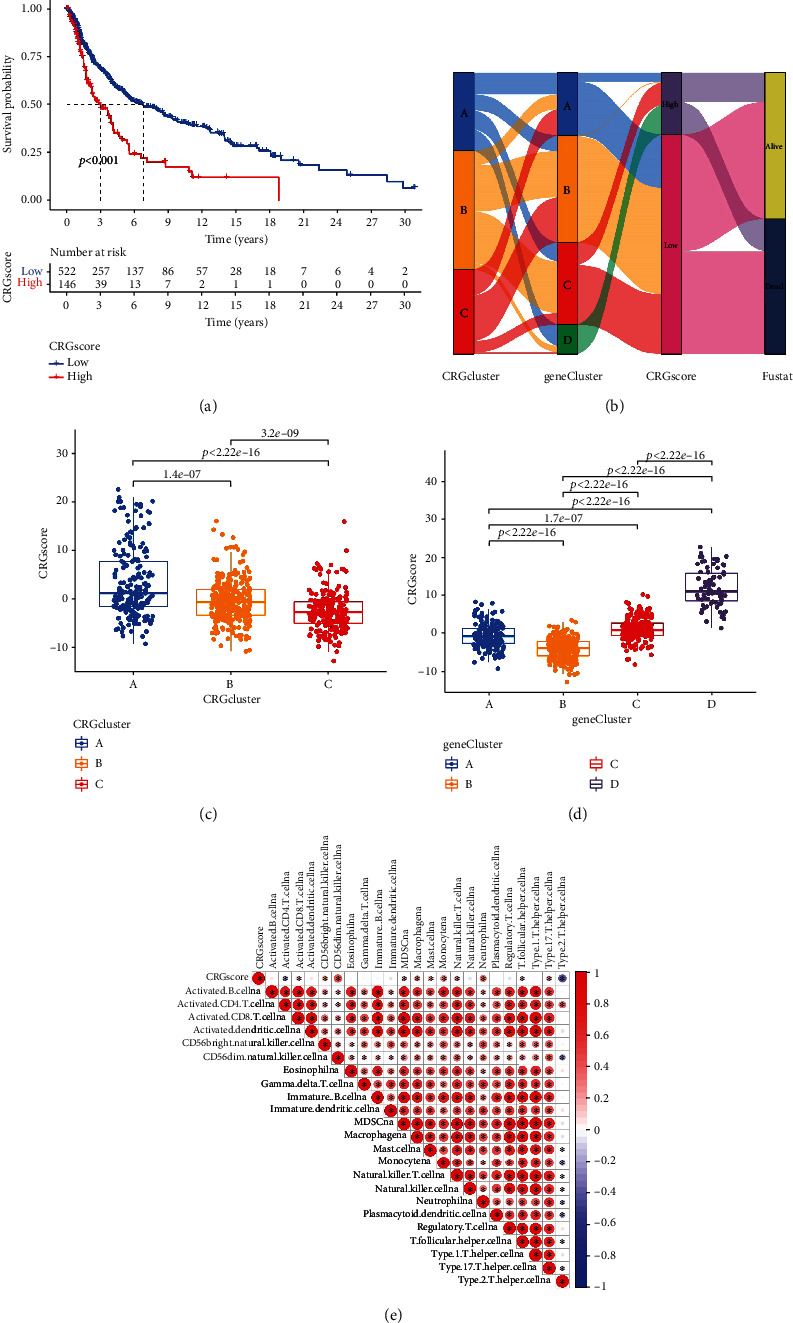
PCA of constructing CRG scores. (a) Survival curves in CM for patients with high and low CRG scores. (b) The Sankey diagrams of the DEG clusters and CRG scores and the distribution of survival states among the various CRG subclusters. (c) Differences in CRG scores between the three CRG subclusters. (d) Differences in CRG scores between the four DEG subclusters. (e) A correlation was found between the CRG scores and the invasion of immune cells in the immunological milieu of the CM. Red indicates a positive connection, whereas blue indicates a negative correlation. ^∗^ indicates a correlation. PCA: principal component analysis; CRG: cuproptosis-related gene; DEGs: differential expressed genes; CM: cutaneous melanoma.

CM patients were given CRG scores 1 and 2 using principal component analysis (PCA), which provided a quantitative assessment of CRG gene association. For CM patients, the total CRG scores 1 and 2 served as independent correlation scores. Ultimately, we came up with a predictive risk score known as the CRG score. Compared to patients with high CRG scores, the survival rate for patients with low CRG scores was much higher (Figure 6(a)). In the meantime, we further illustrated the distribution of CM patients among CRG clusters, gene clusters, CRG score groups, and survival status using a Sankey diagram. The majority of CM patients belonged to the low CRG score group, gene cluster B, and CRG cluster B (Figure 6(b)). Besides, gene cluster D has higher CRG scores, and gene cluster B has lower scores. CRG cluster A possesses higher CRG scores, and gene cluster C possesses lower scores (Figures 6(c) and 6(d)). We examined the relationship between the CRG score and the presence of immune cells to better understand the part that the CRG score plays in the immunotherapy of CM patients. The CRG score correlated positively with CD56 natural killer cells, monocytes, neutrophils, and macrophages, while negatively correlated with the content of activated CD4 T cells and type 2 T helper cells (Figure 6(e)).

### 3.7. Analysis of the Relationship between CRG Scores and Various Clinical Characteristics

**Figure 7:**
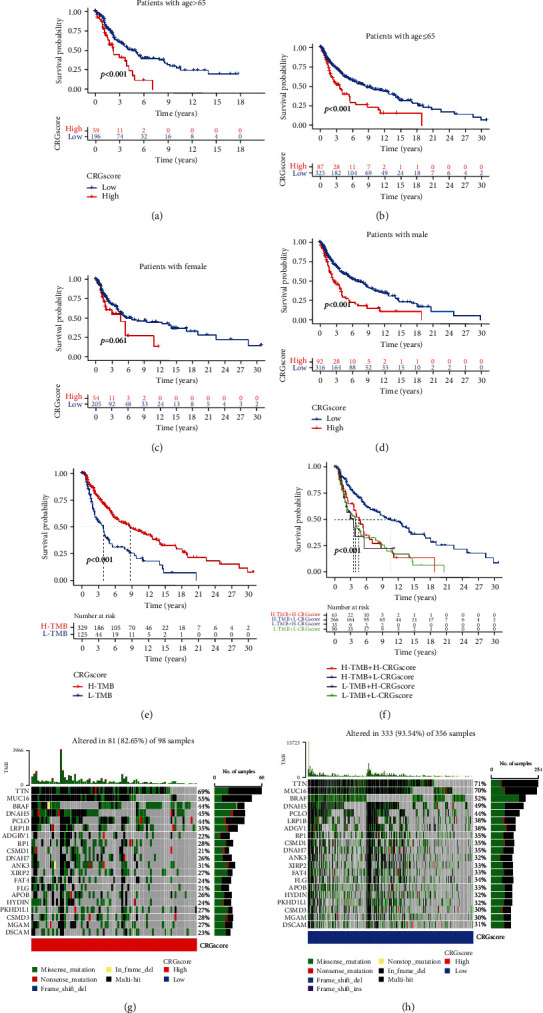
Analysis of the correlation between CRG scores and clinical characteristics. (a–d) K-M survival curves comparing the high CRG score groups to the low CRG score groups across various clinical characteristics. (a) Patients with age > 65. (b) Patients with age ≤ 65. (c) Female patients. (d) Male patients. (e) The K-M method determined survival curves for the high and low TMB groups. (f) K-M curves for CM patients, stratified according to TMB and CRG scores. (g) TMB in the group with high CRG scores. (h) TMB in the group with low CRG scores. CRG: cuproptosis-related gene; TMB: tumor mutation burden; CM: cutaneous melanoma; K-M: Kaplan-Meier.

We broadened research on the association between patient characteristics (age and gender) and the CRG score. Based on many clinical parameters, such as age > 65 and <65, gender, and other factors, we divided CM patients into various subgroups. The OS was better in patients with a low CRG score compared to the high CRG score group in the age and gender subgroups (Figures 7(a)–7(d)). This again implies that the CRG was important for CM patient prognosis and may be a potential biomarker for clinical treatment and CM patient prognostic assessment. In addition, we separated CM patients into groups according to whether their TMB was high or low using the ideal threshold and evaluated the OS in each group. This investigation showed that compared to low TMB, patients with high TMB had better survival (Figure 7(e)). Additionally, patients with high TMB and low CRG scores had a higher OS (Figure 7(f)). This raises the notion that CRG and TMB are connected in some way and that they both have an impact on the prognosis of CM patients. A waterfall diagram was also created to understand better the TMB in patients who were grouped based on their CRG scores. TTN, MUC16, BRAF, DNAH5, and PCLO had the highest mutation frequencies among the top five genes (Figures 7(g) and 7(h)).

### 3.8. The CRG Score's Predictive Value for Immunotherapy Response

**Figure 8:**
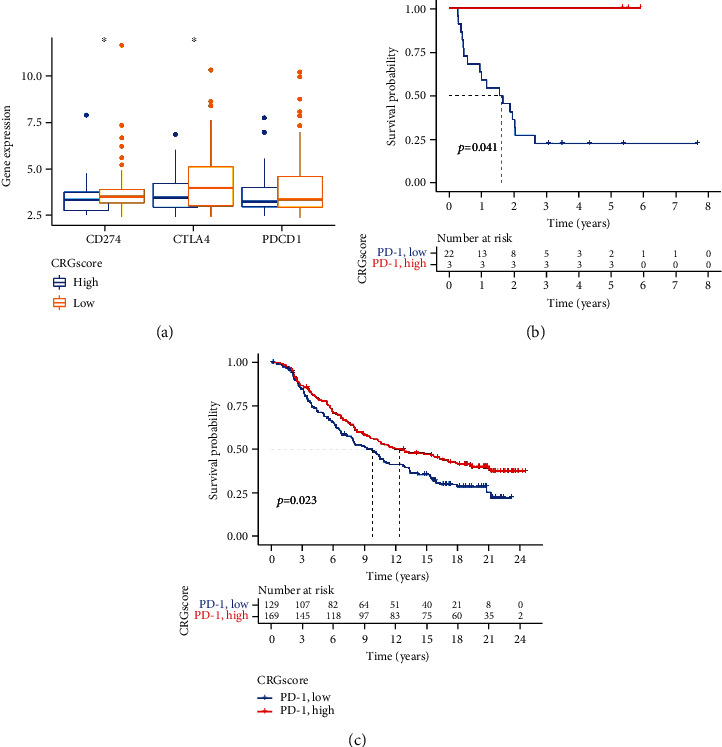
The function of the CRG score in determining the effectiveness of immunotherapy. (a) Variation in ICB gene expression across groups with high and low CRG scores (^∗∗∗^*P* < 0.001 and ^∗∗^*P* < 0.01). (b) Survival curves comparing patients in groups with high and low CRG scores treated with anti-PD-L1 in the GSE93157 cohort. (c) Survival curves comparing patients in groups with high and low CRG scores treated with anti-PD-L1 in the IMvigor210 cohort. CRG: cuproptosis-related gene; ICB: immune checkpoint blocking.

The immunotherapy of malignancies today makes extensive use of ICB genes. One of the key practice mechanisms is the PD-1. However, not all patients respond well to this treatment, and patients with specific malignancies may experience life-threatening immunological adverse effects. We initially examined the expression of ICB genes (PDCD1, CD274, and CTLA4) in the high and low CRG score groups before analyzing the prediction power of the CRG score. We saw a significant uptick in ICB genes' expression in the CRG low score group (Figure 8(a)). Additionally, we gathered two independent cohorts undergoing immunotherapy using an appropriate cutoff and divided them into high and low CRG score groups. According to K-M survival analyses, patients in the GSE93157 cohort who possessed higher CRG scores had longer OS (Figure 8(b)). The IMvigor210 cohort further supported these findings, showing that patients with high CRG scores had a better OS (Figure 8(c)).

## 4. Discussion

Currently, surgery is still the therapy of choice for CM's major emphasis. Tumor immunotherapy, however, offers fresh promise for enhancing the prognosis of patients as a result of the advancement of research into the mechanism of CM and the ongoing development of contemporary biotechnology [30]. Targeted therapy could promote the specific death of tumor cells by analyzing the cell signal transduction pathway and treating the mutated genes of tumor cells and their expression products with antagonistic effects of various drugs.

Copper-induced cell death differs distinctly from traditional death methods (apoptosis, ferroptosis, or necrosis). As a novel cell death pathway, cuprotosis is dependent on mitochondrial respiration—copper causes abnormal aggregation of lipoylated proteins by directly binding to lipoylated proteins in the TAC pathway. And then, it interferes with iron-sulfur cluster proteins in the respiratory chain complex, causing protein toxic stress response, eventually leading to cell death [15]. There are ten known CRG genes, of which seven are positively regulated genes and three negatively regulated genes [31]. Liu et al. found that in PDHA1 knockout (KO) cells, glutamine, glycolysis, and glucose consumption were increased, while oxidative phosphorylation was inhibited. These results indicated that the Warburg effect existed in PDHA1 KO cells. Therefore, inhibition of the expression of the PDHA 1 gene in esophageal squamous cell cancer (ESCC) may lead to the Warburg effect of metabolic reprogramming and the increase of malignant tumors [32]. Ji et al. reported that a poor prognosis and disease recurrence were associated with high expression of MTF1, which was upregulated in ovarian cancer. MTF1 has cancer-causing actions and encourages EMT, which aids in the spread of ovarian tumors [33]. Chen et al. explained that LIPT1 could inhibit T24 cell migration to a certain extent in urothelial carcinoma and was related to cell proliferation and migration [34]. GLS was discovered to be a novel oncogene in prostate cancer by Zhang et al. They also discovered that GLS was markedly elevated in prostate cancer tissues and cell lines. GLS silencing significantly reduced the ability of DU145 and PC-3 cells to proliferate. Cell cycle arrest and apoptosis were triggered by low GLS expression [35]. Zhu et al. showed that miR-146b-5p targeted the 3′-UTR of PDHB and is carcinogenic. Overexpression of PDHB can reverse miR-146b-5p carcinogenic effects on the development, invasion, and glycolysis of colorectal cancer (CRC) cells. In CRC, miR-146b-5p was used to specifically target PDHB and exert its oncogenic effect [36]. According to research by Zda et al., LIAS and RPL9 interact and aberrant expression of both genes may be linked to the development of lung cancer [37]. By reducing the ability of DLD-1 cells to invade through the AMPK/integrin/FAK axis, Huang et al. provided evidence that adenine may have antimetastasis potential in CRC cells [38]. In 1994, the gene for the cell cycle inhibitor, CDKN2A, was discovered. Since then, numerous cancers have been linked to somatic mutations, and close relatives of people with familial atypical multiple mole/melanoma have been found to have altered germ cells [39]. FDX1 is the most critical regulator of cuproptosis. FDX1 is highly associated with the expression of lipoacylated protein. And FDX1 gene KO resulted in complete loss of protein lipoacylated [40]. DLAT was a subunit of pyruvate dehydrogenase complex, and siRNA studies supported the role of DLAT in gastric cancer proliferation and carbohydrate metabolism [41].

In this study, we developed three different subclusters of CRG in CM (CRG clusters 1, 2, and 3) using TCGA and GSE65904 cohort data. Then, the pairwise difference analysis of the three groups was carried out, and 421 differential genes were obtained after the intersection. The results of GO enrichment analysis of intersecting genes show differences among the three groups mainly in the biological processes of the cell cycle, DNA replication, and nucleotide excision and repair, which may be the reason for the differences in the occurrence of copper death. GSVA shows differences in ascorbic acid and alcoholic acid metabolism, olfactory conduction, protein output, glyoxylic acid and dicarboxylic acid metabolism, citric acid cycle, and TCA cycle among the three groups of clusters 1, 2, and 3, respectively. Then, we continue to use the unsupervised clustering method for intersection genes and get four DEG subclusters. There was no difference in OS among the three CRG subclusters, but there were significant differences in OS of DEG subclusters among the three CRG subclusters. To further assess the CRG score's ability to predict immunotherapy response, we also developed a quantitative scoring system known as the CRG score. Besides, TMB can predict the effectiveness of immunotherapy for different tumor types and indirectly reflect the tumor's capacity and level of neoantigen generation. TMB is a biomarker for ICI efficacy prediction and can predict PD-1 and CTLA-4 antibody treatment responses. In 2014, Snyder et al., for the first time, found a correlation between the efficacy of TMB and anti-CTLA-4 antibody in melanoma [42]. The top 5 genes with the highest mutation frequency in our analysis, whether high or low CRG score groups, were TTN, MUC16, BRAF, DNAH5, and PCLO. It has been reported that CDKN2A will be deleted in metastatic melanoma. At the same time, the mutation rate of TTN and MUC16 will increase in patients with CDKN2A deletion, which suggests that the increased mutation of TTN and MUC16 in this study may lead to metastasis of melanoma [43]. Currently, the targeted therapy of BRAF has become the first-line treatment scheme for skin melanoma with advanced BRAF mutation. The BRAF mutation in the high and low CRG score groups is increased, which provides a solution for its treatment [44]. Recently, DNAH5 has been reported to have a mutation in melanoma, which has an important influence on melanoma development [45]. However, PCLO has not been reported in melanoma. It has been found that there are mutations in patients with schizophrenia, bipolar disorder, and major depression [46]. In addition, in the copy number change frequency analysis, the probability of loss of the copy of the CDKN2A gene in CRG was significantly higher than that of a gain copy. This evidence suggested that CRG was associated with the stabilization and significant accumulation of mutations in TME and may be a prognostic marker for melanoma patients. However, given the individual differences between TME and clinical patients, PCA analysis was used to construct CRG scores to distinguish melanoma patients quantitatively. Compared with the high CRG score group, the low CRG score group had better OS. Moreover, the high TMB + low CRG score group had the best OS. GSEA suggested that CRG score positively correlated with CD56 natural killer cells (NK cells), monocytes, neutrophils, and macrophages but negatively correlated with activated CD4 T cells and type 2 T helper cells. Melanoma can migrate to the liver, lungs, and other organs when it starts to grow. Cells in the immune system are constantly watching our body tissues. T helper cells help dendritic cells activate cytotoxic T cells, release cytokines like interferon, and attract more NK cells, which release stress-related factors to these cancer cells. By identifying the antigens made by HLA class II molecules in the tumor microenvironment, CD4 T cells can encourage antitumor responses. By detecting HLA I-restricted tumor-related antigens and HLA II-restricted neoantigens, melanoma cells can directly activate depleted cytotoxic CD4+T cells. Antigen-presenting cells showed tumor antigens to CD4 T regulatory (Treg) cells, which indirectly made CD4 T regulatory (Treg) cells. When neoantigen loading in tumors was exceptionally high, this effect was seen [47]. This may help to explain why the low CRG score group had a better prognosis. CRG score has different biological effects from TMB, which can guide immunotherapy.

We discovered substantial differences in the levels of certain ICB genes (CD274, CDLA4, and PDCD1) between the high and low CRG score groups while testing the efficacy of CRG score for immunotherapy. ICB gene expression was higher in the low CRG score group, suggesting that CM patients with low CRG scores might benefit more from immunotherapy. The regulatory factor PD-1, which is found on the surface of T cells, inhibits T cell activation. The overexpression of PD-L1 and PD-L2 was detected in various malignant tumors. The interaction of PD-1 and its ligand PD-L1 can effectively inhibit the activation of T cell antigen receptors and then play an inhibitory effect on the activation and proliferation of T lymphocytes. CTLA-4 is a negative regulator of T cell activation, which can induce T cell nonreactivity and exert a corresponding immunosuppressive effect. The interaction of the two mechanisms above causes the immune escape of tumor cells, which makes tumor cells escape the attack of the body's immune system. It then promotes the occurrence and development of tumors. Such immune checkpoint has become an important direction of antitumor immunotherapy. However, after the evaluation of immunotherapy for CM patients, we found that when anti-CTLA4 and anti-PDL1/PD1 were used, the immunotherapy effect of the CRG score groups was not statistically significant. Therefore, we further validated this with two samples treated with anti-PD-1/PD-L1. Results showed that the high CRG score groups had a better prognosis after receiving immunotherapy. This was contrary to our previous hypothesis. It may be caused that the samples of these two cohorts were not all CM but included other types of carcinoma. However, it could still reflect that CRG score was related to prognosis after receiving immunotherapy.

## 5. Conclusions

In a word, we use unsupervised clustering and PCA algorithm to analyze the TCGA database and develop a CRG scoring system for CM and verify the credibility of CRG score on OS and TMB through external dataset GSE65904, which provides a new perspective and idea for evaluating the prognosis of CM patients and the effectiveness of immunotherapy in the future.

## Data Availability

The data used to support the findings of this study are included in the article.

## References

[1] Strashilov S., Yordanov A. (2021). Aetiology and pathogenesis of cutaneous melanoma: current concepts and advances. *International Journal of Molecular Sciences*.

[2] Sayan M., Mamidanna S., Oncel D., Jan I., Chundury A. (2020). Clinical Management of Uveal Melanoma: A Comprehensive Review with a Treatment Algorithm. *Radiation Oncology Journal*.

[3] Chang A. E., Karnell L. H., Menck H. R., The American College of Surgeons Commission on Cancer and the American Cancer Society (2015). The National Cancer Data Base report on cutaneous and noncutaneous melanoma: a summary of 84,836 cases from the past decade. *Cancer*.

[4] Siegel R. L., Miller K. D., Fuchs H. E., Jemal A. (2022). Cancer statistics, 2022. *CA: a Cancer Journal for Clinicians*.

[5] Ekwueme D. U., Guy G. P., Li C., Sun H. R., Parelkar P., Chen S. C. (2011). The health burden and economic costs of cutaneous melanoma mortality by race/ethnicity–United States,2000 to 2006. *Journal of the American Academy of Dermatology*.

[6] Whiteman D., Green A. (1999). The pathogenesis of melanoma induced by ultraviolet radiation. *New England Journal of Medicine*.

[7] Luca F., Andrea M., Carla L., Sabrina F., Demetrios A. (2016). Spandidos: Occupational exposure to carcinogens: benzene, pesticides and fibers (review). *Molecular Medicine Reports*.

[8] Authors M., Merlin J. L. (2013). Signatures of Mutational Processes in Human Cancer. *Nature*.

[9] Cheng Y., Zhang G., Li G. (2013). Targeting MAPK pathway in melanoma therapy. *Cancer Metastasis Reviews*.

[10] Curti B. D., Faries M. B. (2021). Recent advances in the treatment of melanoma. *The New England Journal of Medicine*.

[11] Schäfer A., Haenig B., Erupathil J. (2021). Inhibition of endothelin-B receptor signaling synergizes with MAPK pathway inhibitors in BRAF mutated melanoma. *Oncogene*.

[12] Bhatia S., Tykodi S. S., Lee S. M., Thompson J. A. (2015). Systemic therapy of metastatic melanoma: on the road to cure. *Oncology*.

[13] Gottschlich A., Endres S., Kobold S. (2021). Therapeutic strategies for targeting IL-1 in cancer. *Cancers (Basel)*.

[14] Sim F. H., Taylor W. F., Ivins J. C., Pritchard D. J., Soule E. H. (2015). A prospective randomized study of the efficacy of routine elective lymphadenectomy in management of malignant melanoma. *Preliminary Results. Cancer*.

[15] Jiang Y., Huo Z., Qi X., Zuo T., Wu Z. (2022). Copper-induced tumor cell death mechanisms and anti-tumor theragnostic applications of copper complexes. *Nanomedicine (London, England)*.

[16] Tang D., Chen X., Kroemer G. (2022). Cuproptosis: a copper-triggered modality of mitochondrial cell death. *Cell Research*.

[17] Tsvetkov P., Coy S., Petrova B. (2022). Copper induces cell death by targeting lipoylated TCA cycle proteins. *Science*.

[18] Bian Z., Fan R., Xie L. (2022). A novel cuproptosis-related prognostic gene signature and validation of differential expression in clear cell renal cell carcinoma. *Genes (Basel)*.

[19] Wu C., Tan J., Wang X. (2022). *Pan-Cancer Analyses Reveal Molecular And Clinical Characteristics Of Cuproptosis Regulators In Cancer*.

[20] Lelièvre P., Sancey L., Coll J. L., Deniaud A., Busser B. (2020). The multifaceted roles of copper in cancer: a trace metal element with dysregulated metabolism, but also a target or a bullet for therapy. *Cancers (Basel)*.

[21] Li S. R., Bu L. L., Cai L. (2022). Cuproptosis: lipoylated TCA cycle proteins-mediated novel cell death pathway. *Signal Transduction and Targeted Therapy*.

[22] Wagner G. P., Kin K., Lynch V. J. (2012). Measurement of mRNA abundance using RNA-seq data: RPKM measure is inconsistent among samples. *Theory in Biosciences*.

[23] Hänzelmann S., Castelo R., Guinney J. (2013). GSVA: the gene set variation analysis package for microarray and RNA-seq data. *BMC Bioinformatics*.

[24] Ashburner M., Ball C. A., Blake J. A. (2000). Gene ontology: tool for the unification of biology. The gene ontology consortium. *Nature Genetics*.

[25] Kanehisa G., Goto S. (2000). KEGG: Kyoto Encyclopedia of Genes and Genomes. *Nucleic Acids Research*.

[26] Mayakonda A., Lin D. C., Assenov Y., Plass C., Koeffler H. P. (2018). Maftools: efficient and comprehensive analysis of somatic variants in cancer. *Genome Research*.

[27] Hugo W., Zaretsky J., Sun L. (2016). Genomic and transcriptomic features of response to anti-PD-1 therapy in metastatic melanoma. *Cell*.

[28] Ayers M., Lunceford J., Nebozhyn M. (2017). IFN-*γ*–related mRNA profile predicts clinical response to PD-1 blockade. *The Journal of Clinical Investigation*.

[29] Meng J., Huang X., Qiu Y., Yu M., Lu J., Yao J. (2021). Characterization of m6A-related genes landscape in skin cutaneous melanoma to aid immunotherapy and assess prognosis. *International Journal of General Medicine*.

[30] Luke J. J., Flaherty K. T., Ribas A., Long G. V. (2017). Targeted agents and immunotherapies: optimizing outcomes in melanoma. *Nature Reviews Clinical Oncology*.

[31] Jia L.-Y., Bai J.-Y., Sun K., Wang R. F., Feng H. Q. (2019). Extracellular ATP released by copper stress could act as diffusible signal in alleviating the copper stress-induced cell death. *Protoplasma*.

[32] Liu L., Cao J., Zhao J., Li X., Suo Z., Li H. (2019). PDHA1 gene knockout in human esophageal squamous cancer cells resulted in greater Warburg effect and aggressive features in vitro and in vivo. *Oncotargets and Therapy*.

[33] Ji L., Zhao G., Zhang P. (2018). Knockout of MTF1 inhibits the epithelial to mesenchymal transition in ovarian cancer cells. *Journal of Cancer*.

[34] Chen Y., Xu T., Xie F., Wang L., Jiao W. (2021). Evaluating the biological functions of the prognostic genes identified by the Pathology Atlas in bladder cancer. *Oncology Reports*.

[35] Zhang J., Mao S. Y., Guo Y. D., Wu Y., Yao X. D., Huang Y. (2019). Inhibition of GLS suppresses proliferation and promotes apoptosis in prostate cancer. *Bioscience Reports*.

[36] Zhu Y., Wu G., Yan W., Zhan H., Sun P. (2017). miR-146b-5p regulates cell growth, invasion, and metabolism by targeting PDHB in colorectal cancer. *American Journal of Cancer Research*.

[37] Dlamini Z., Marima R., Hull R. (2021). Genomics and molecular analysis of Rpl9 and lias in lung cancer: emerging implications in carcinogenesis. *Informatics in Medicine Unlocked*.

[38] Huang C. W., Lin Y. C., Hung C. H. (2021). Adenine inhibits the invasive potential of DLD-1 human colorectal cancer cell via the AMPK/FAK axis. *Pharmaceuticals (Basel)*.

[39] Foulkes W. D., Flanders T. Y., Pollock P. M., Hayward N. K. (1997). The CDKN2A (p16) gene and human cancer. *Molecular Medicine*.

[40] Zhang Z., Ma Y., Guo X. (2021). FDX1 can impact the prognosis and mediate the metabolism of lung adenocarcinoma. *Frontiers in Pharmacology*.

[41] Wen Q., Ow G. S., Kuznetsov V. A., Chong S., Lim Y. P. (2015). DLAT subunit of the pyruvate dehydrogenase complex is up-regulated in gastric cancer-implications in cancer therapy. *American Journal of Translational Research*.

[42] Snyder A., Makarov V., Merghoub T. (2014). Genetic basis for clinical response to CTLA-4 blockade in melanoma. *The New England Journal of Medicine*.

[43] Arnoff T. E., El-Deiry W. S. (2022). MDM2/MDM4 amplification and CDKN2A deletion in metastatic melanoma and glioblastoma multiforme may have implications for targeted therapeutics and immunotherapy. *American Journal of Cancer Research*.

[44] Pavlick A. C., Fecher L., Ascierto P. A., Sullivan R. J. (2019). Frontline therapy for BRAF-mutated metastatic melanoma: how do you choose, and is there one correct answer?. *American Society of Clinical Oncology Educational Book*.

[45] Zhang D., Xia J. (2020). Somatic synonymous mutations in regulatory elements contribute to the genetic aetiology of melanoma. *BMC Medical Genomics*.

[46] Chen C. H., Huang Y. S., Liao D. L., Huang C. Y., Lin C. H., Fang T. H. (2021). Identification of rare mutations of two presynaptic cytomatrix genes BSN and PCLO in schizophrenia and bipolar disorder. *Journal of Personalized Medicine*.

[47] Oliveira G., Stromhaug K., Cieri N. (2022). Landscape of helper and regulatory antitumour CD4^+^ T cells in melanoma. *Nature*.

